# Electrochemical Biosensors for the Detection of Exosomal microRNA Biomarkers for Early Diagnosis of Neurodegenerative Diseases

**DOI:** 10.1021/acs.analchem.4c02619

**Published:** 2025-03-09

**Authors:** Jiacheng Yu, Runzhi Zhou, Shan Liu, Jintao Zheng, Haoyang Yan, Song Su, Ningli Chai, Ester Segal, Cheng Jiang, Keying Guo, Chen-zhong Li

**Affiliations:** † Biotechnology and Food Engineering, Guangdong Technion-Israel Institute of Technology (GTIIT), Shantou 515063, China; ‡ Faculty of Biotechnology and Food Engineering, Technion-Israel Institute of Technology (IIT), Haifa 3200003, Israel; § Sichuan Provincial Key Laboratory for Human Disease Gene Study, Department of Medical Genetics, Sichuan Academy of Medical Sciences & Sichuan Provincial People’s Hospital, University of Electronic Science and Technology of China, Chengdu 610072, China; ∥ Department of Gastroenterology, The First Medical Center of Chinese PLA General Hospital, Beijing 100853, China; ⊥ School of Medicine, The Chinese University of Hong Kong Shenzhen, Shenzhen 518172, China; # Guangdong Provincial Key Laboratory of Materials and Technologies for Energy Conversion, Shantou 515063, China; g Monash Institute of Pharmaceutical Sciences (MIPS), Monash University, Parkville VIC 3052, Australia

## Abstract

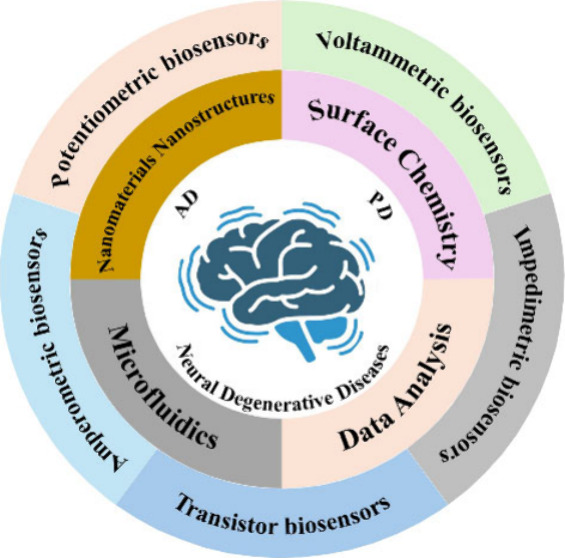

Early and precise diagnosis of neurodegenerative disorders like Alzheimer’s (AD) and Parkinson’s (PD) is crucial for slowing their progression and enhancing patient outcomes. Exosomal microRNAs (miRNAs) are emerging as promising biomarkers due to their ability to reflect the diseases’ pathology, yet their low abundance poses significant detection hurdles. This review article delves into the burgeoning field of electrochemical biosensors, designed for the precise detection of exosomal miRNA biomarkers. Electrochemical biosensors offer a compelling solution, combining the sensitivity required to detect low-abundance biomarkers with the specificity needed to discern miRNA profiles distinctive to neural pathological states. We explore the operational principles of these biosensors, including the electrochemical transduction mechanisms that facilitate miRNA detection. The review also summarizes advancements in nanotechnology, signal enhancement, bioreceptor anchoring, and microfluidic integration that improve sensor accuracy. The evidence of their use in neurodegenerative disease diagnosis is analyzed, focusing on the clinical impact, diagnostic precision, and obstacles faced in practical applications. Their potential integration into point-of-care testing and regulatory considerations for their market entry are discussed. Looking toward the future, the article highlights forthcoming innovations that might revolutionize early diagnostic processes. Electrochemical biosensors, with their impressive sensitivity, specificity, and point-of-care compatibility, are on track to become instrumental in the early diagnosis of neurodegenerative diseases, possibly transforming patient care and prognosis.

## Introduction

Neurodegenerative diseases (NDD), such as Alzheimer’s disease (AD) and Parkinson’s disease (PD), present a significant global health burden due to their progressive nature and the lack of effective treatments.^1−6^ Early diagnosis of these diseases is crucial as it provides an opportunity for timely interventions that may slow down disease progression and improve patient outcomes.^7−11^ However, achieving early diagnosis poses several challenges, including the subtle onset of symptoms and the overlap of clinical manifestations with other conditions.^12−15^ To overcome these challenges, researchers have turned to utilizing biomarkers as potential diagnostic targets.^16^

Biomarkers are measurable indicators of biological processes or disease states.^17−20^ They provide valuable insights into disease progression, response to treatment, and prognosis.^21−24^ Among the various biomarkers investigated, microRNAs (miRNAs) have gained attention due to their role in regulating gene expression and their potential as indicators of neural degeneration.^25−27^ In particular, miRNAs encapsulated within exosomes, small extracellular vesicles involved in intercellular communication, have shown promise for their stability and ability to reflect disease-specific changes.^28−31^

The identification and detection of exosomal miRNA biomarkers have opened new possibilities in the early diagnosis of neurodegenerative diseases.^32−34^ By analyzing the distinct miRNA profiles found in exosomes, researchers can gain insights into the underlying molecular mechanisms and pathophysiological changes associated with these diseases.^35,36^ However, the detection of exosomal miRNAs presents unique technical challenges due to their low abundance in biological fluids and the need for sensitive and specific detection methods.^37,38^

Herein, we focus on the potential of electrochemical biosensors as a promising technology for detecting exosomal miRNA biomarkers in the context of the early diagnosis of neurodegenerative diseases. Electrochemical biosensors offer several advantages, including high sensitivity, rapid response, cost-effectiveness, and the potential for point-of-care applications. These biosensors operate by converting biochemical interactions between exosomal miRNAs and specific recognition elements into measurable electrical signals.

**Figure 1:**
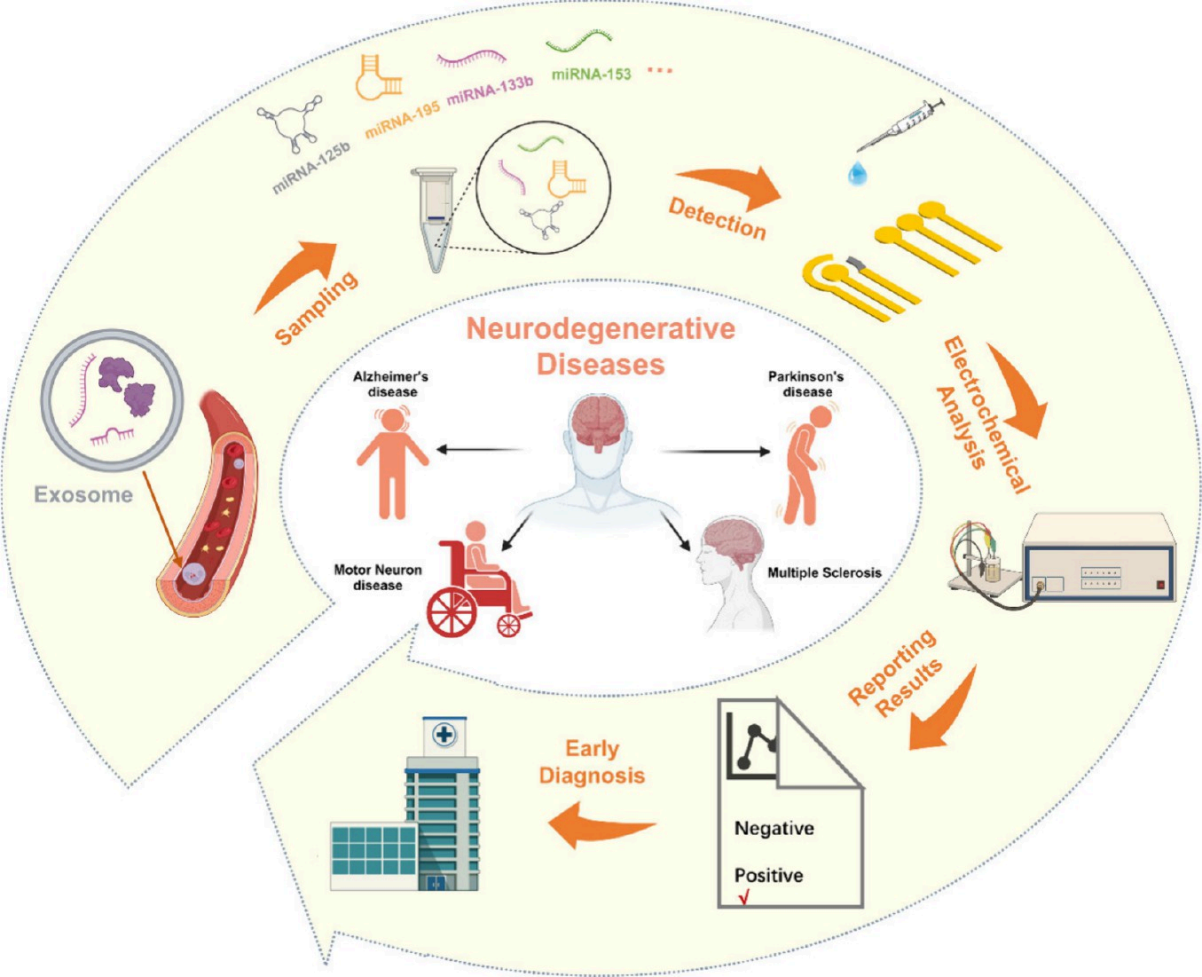
Schematic illustration of the electrochemical biosensors’ detection of exosomal miRNA biomarkers for the early diagnosis of neurodegenerative diseases.

In this Review, we provide a comprehensive overview of the background and significance of early diagnosis in neurodegenerative diseases. We discuss the challenges associated with current diagnostic approaches and highlight the need for reliable biomarkers. Specifically, we delve into the role of exosomal miRNAs as potential biomarkers and their advantages over other types of biomarkers. Additionally, we introduce the concept of electrochemical biosensors and their relevance in the detection of exosomal miRNA biomarkers (Figure 1). We explore the principles of electrochemical biosensors and their potential to revolutionize early diagnosis by providing the sensitive and specific detection of exosomal miRNAs.

## Exosomal MicroRNAs as Biomarkers

Exosomes, small extracellular vesicles released by various cell types, have garnered considerable attention as carriers of biological molecules, including microRNAs (miRNAs).^39−41^ miRNAs are short noncoding RNA molecules that play critical roles in post-transcriptional gene regulation, influencing a wide range of biological processes.^42,43^

Exosomal miRNAs have emerged as promising biomarkers for neurodegenerative diseases, owing to their unique characteristics.^44−46^ The biology of exosomal miRNAs offers several advantages over other biomarkers, making them attractive for early diagnosis and disease monitoring.^47,48^ For example, exosomal miRNAs like miRNA-200, miRNA-141, miRNA-122, and miRNA-32 have been reported as biomarkers for ovarian,^49^ colon,^50^ liver,^51^ and prostate tumors,^52^ respectively.

First, exosomes provide protection and stability to miRNAs.^53^ Encased within the lipid bilayer, miRNAs are shielded from enzymatic degradation and other environmental factors.^54,55^ This protection ensures that exosomal miRNAs remain intact and detectable in various biological fluids, such as blood, cerebrospinal fluid (CSF), and urine.^56^ Consequently, the stability of exosomal miRNAs allows for noninvasive sample collection and facilitates their potential clinical utility as diagnostic markers.

Second, the specific distribution of miRNAs in exosomes provides valuable insights into neurodegenerative diseases.^57,58^ The packaging of miRNAs into exosomes is a tightly regulated process, influenced by both cellular and disease-specific factors.^59^ The unique miRNA profiles found in exosomes from patients with neurodegenerative diseases reflect the underlying pathophysiological changes occurring in affected neural tissues.^60−62^ These disease-specific miRNA signatures offer a glimpse into disease progression, severity, and response to treatment.^63^ By analyzing the differential expression of exosomal miRNAs, researchers can potentially identify specific biomarker panels that distinguish between healthy individuals and those with neurodegenerative diseases.

Moreover, exosomal miRNAs have been implicated in the intercellular communication network involved in neurodegenerative diseases.^64^ Exosomes can transfer miRNAs from donor cells to recipient cells, thereby influencing gene expression and signaling pathways in recipient cells.^65^ This transfer of miRNAs between cells in the central nervous system enables the spread of pathological changes, contributing to disease progression.^66^ The ability of exosomal miRNAs to act as signaling molecules makes them valuable biomarkers for understanding disease mechanisms and potentially developing targeted therapeutic interventions.^67^

Compared to other biomarkers, such as protein or genetic markers, exosomal miRNAs offer several advantages.^68,69^ They are noninvasive and easily detectable and exhibit robust stability in various biological fluids.^70^ The ability to detect disease-specific miRNA profiles in exosomes provides a unique and dynamic window.^71^

## Electrochemical Biosensors: Principles and Mechanisms

Electrochemical biosensors have emerged as powerful tools for the detection of exosomal microRNA (miRNA) biomarkers in neurodegenerative diseases.^3,72,73^ These biosensors operate on the fundamental principle of converting biochemical interactions between target analytes and specific recognition elements into measurable electrical signals.^74,75^ This section provides an overview of the basic principles of electrochemical biosensors and highlights the different types of electrochemical sensors commonly employed for miRNA detection. Additionally, specific transduction mechanisms relevant to miRNA detection are discussed.

The basic principle of electrochemical biosensors involves the measurement of electrical properties resulting from biochemical interactions at the sensing interface. These biosensors consist of three essential components: the recognition element, the transducer, and the signal processing system. The recognition element is typically a bioreceptor molecule, such as a nucleic acid, antibody, or aptamer, which specifically binds to the target miRNA.^76−78^ The transducer converts the biochemical recognition event into an electrical signal. Finally, the signal processing system amplifies and analyzes the electrical signal to provide quantification or identification of the target miRNA.^76^

Various types of electrochemical biosensors have been developed for miRNA detection, with each employing different measurement principles and electrode configurations. Some commonly used types include the following.

### Potentiometric Biosensors

**Figure 2:**
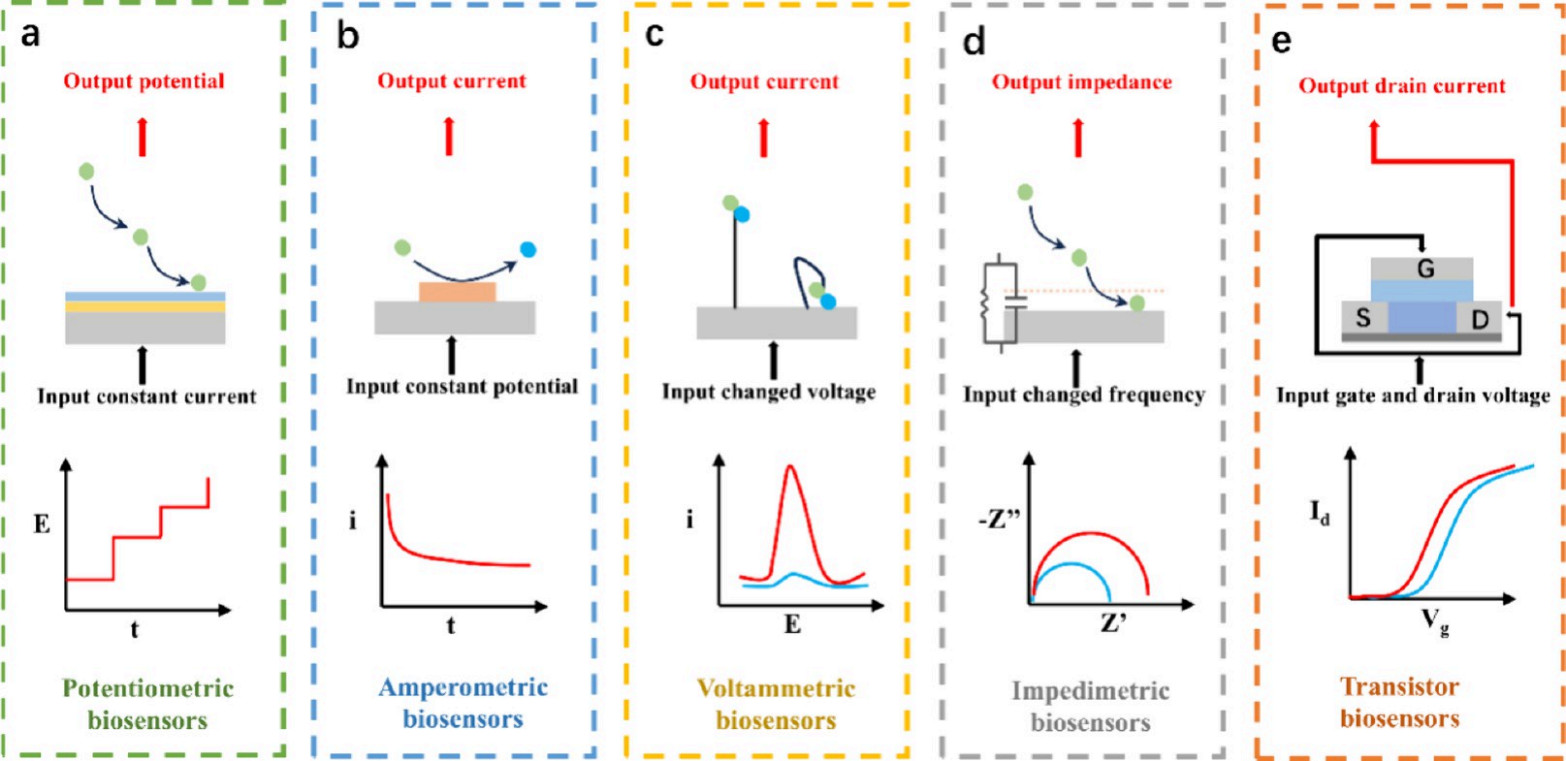
Principles and mechanisms of electrochemical biosensors. a) Potentiometric biosensors, b) amperometric biosensors, c) voltammetric biosensors, d) impedimetric biosensors, and e) transistor biosensors.

These biosensors measure the potential difference generated at the electrode–solution interface due to the binding events occurring on the electrode surface.^79^ Changes in potential are detected using ion-selective electrodes or solid-state junctions, providing a direct readout of miRNA binding (Figure 2a).

### Amperometric Biosensors

In amperometric biosensors,^80^ an applied potential is used to induce an oxidation or reduction reaction involving the target miRNA or a redox probe. The resulting current is measured and correlated to the concentration of the miRNA. Enzymatic amplification strategies are often employed to enhance the sensitivity and selectivity of amperometric biosensors (Figure 2b).

### Voltammetric Biosensors

Voltammetric biosensors employ a potential sweep across the working electrode, producing a current response that is measured as a function of the applied potential.^81^ Cyclic voltammetry, differential pulse voltammetry, and square wave voltammetry are commonly employed techniques. The shape and magnitude of the resulting current response provide information about the miRNA concentration (Figure 2c).

### Impedimetric Biosensors

As shown in Figure 2d, impedimetric biosensors measure changes in the electrical impedance at the electrode–electrolyte interface due to the binding events.^82^ In general, the binding of biomarkers (miRNA) on the electrode surface will cause changes in the capacitance, resistance, or inductance of the electric double layer on the electrode surface, and the changes in the electrical data related to miRNA can be obtained by measuring the impedance and reasonable data fitting processing so as to achieve a qualitative or quantitative analysis of miRNA.^83^

### Transistor Biosensors

Transistor biosensors are the amplification of electrical signals through transistors (voltage-controlled current source devices).^84−86^ Transistor biosensors benefit from easy fabrication, high sensitivity, flexibility, and low energy consumption. Common transistor biosensors include metal oxide semiconductor field-effect transistor (MOSFET) biosensors,^87^ organic field-effect transistor (OFET) biosensors,^88^ organic thin-film transistor (OTFT) biosensors,^89^ organic electrochemical transistor (OECT) biosensors,^90−92^ etc. (Figure 2e).

## Advances in Electrochemical Biosensor Technology for Exosomal MicroRNA Detection

The field of electrochemical biosensors has seen remarkable advances, particularly in their application to the detection of exosomal microRNA (miRNA) biomarkers. Innovations such as the incorporation of functional nanomaterials, tuning surface chemistry, and integration using microfluidics have enhanced the sensitivity, specificity, and practicality of biosensors, making early diagnosis of neurodegenerative diseases increasingly attainable. This section discusses the latest advancements in electrochemical biosensor technology, focusing on these designs and applications in exosomal miRNA detection.

### Nanomaterials and Nanostructures for Signal Amplification

**Figure 3:**
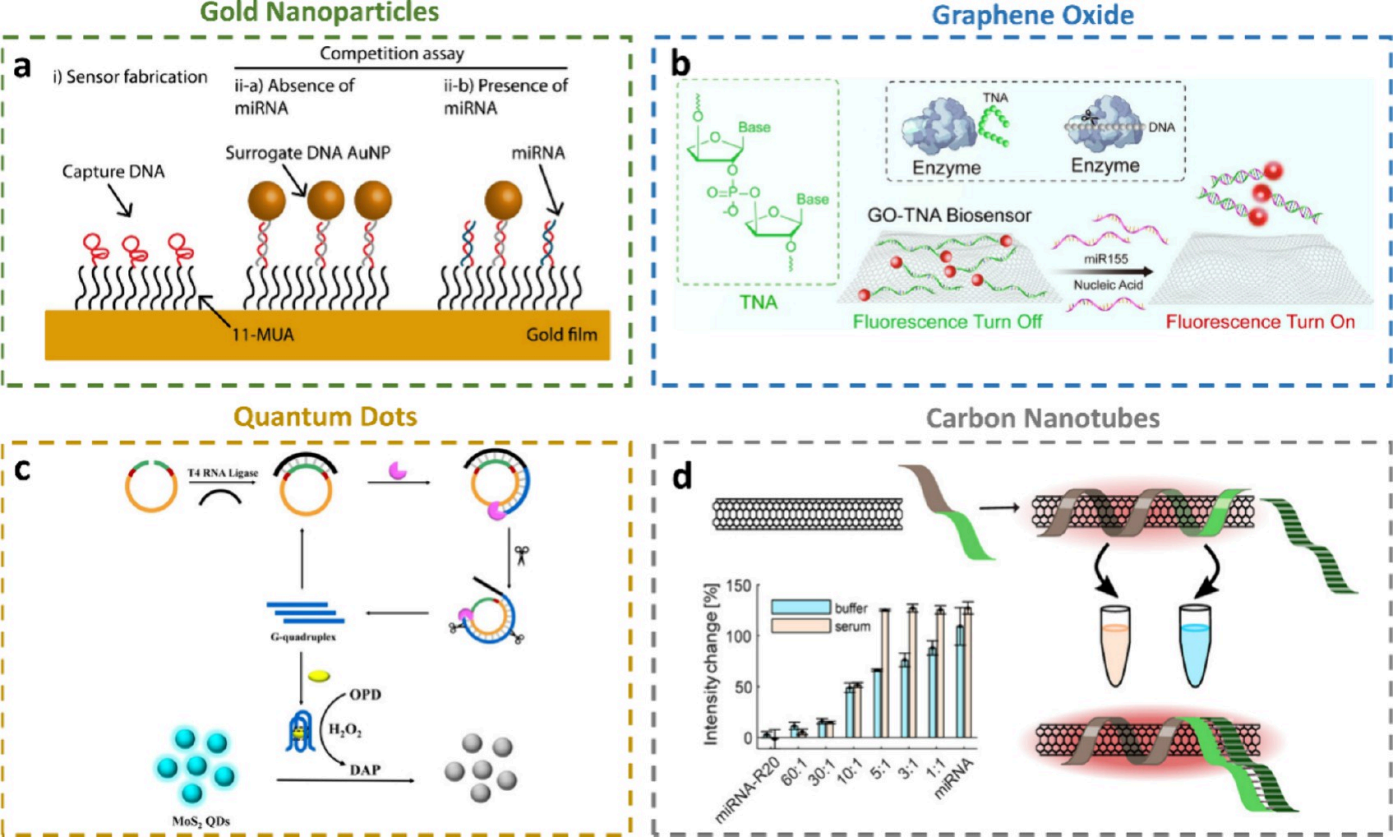
Nanomaterials and nanostructures for signal amplification. a) Gold nanoparticles for signal amplification. Reprinted from ref (93). Copyright 2018, with permission from Elsevier. b) Graphene oxide for signal amplification. Reprinted from ref (94). Copyright 2024, with permission from Elsevier. c) Quantum dots for signal amplification. Reprinted from ref (95). Copyright 2020 American Chemical Society. d) Carbon nanotubes for signal amplification. Reprinted from ref (96). Copyright 2023 American Chemical Society.

Nanomaterials have been pivotal in advancing electrochemical biosensors for miRNA detection. Due to their high surface-to-volume ratio and exceptional electrical, thermal, and catalytic properties, nanomaterials can significantly amplify signals. Materials such as gold nanoparticles,^93^ graphene oxide,^94^ quantum dots,^95^ and carbon nanotubes^96^ are regularly employed for this purpose (Figure 3). These materials offer a larger surface area for bioreceptor immobilization, enhancing sensitivity and enabling the detection of low-abundance exosomal miRNAs. They also facilitate quicker electron transfer between the recognition elements and the electrodes, which is crucial for producing a rapid and robust signal.

### Surface Chemistry and Bioreceptor Immobilization Techniques

**Figure 4:**
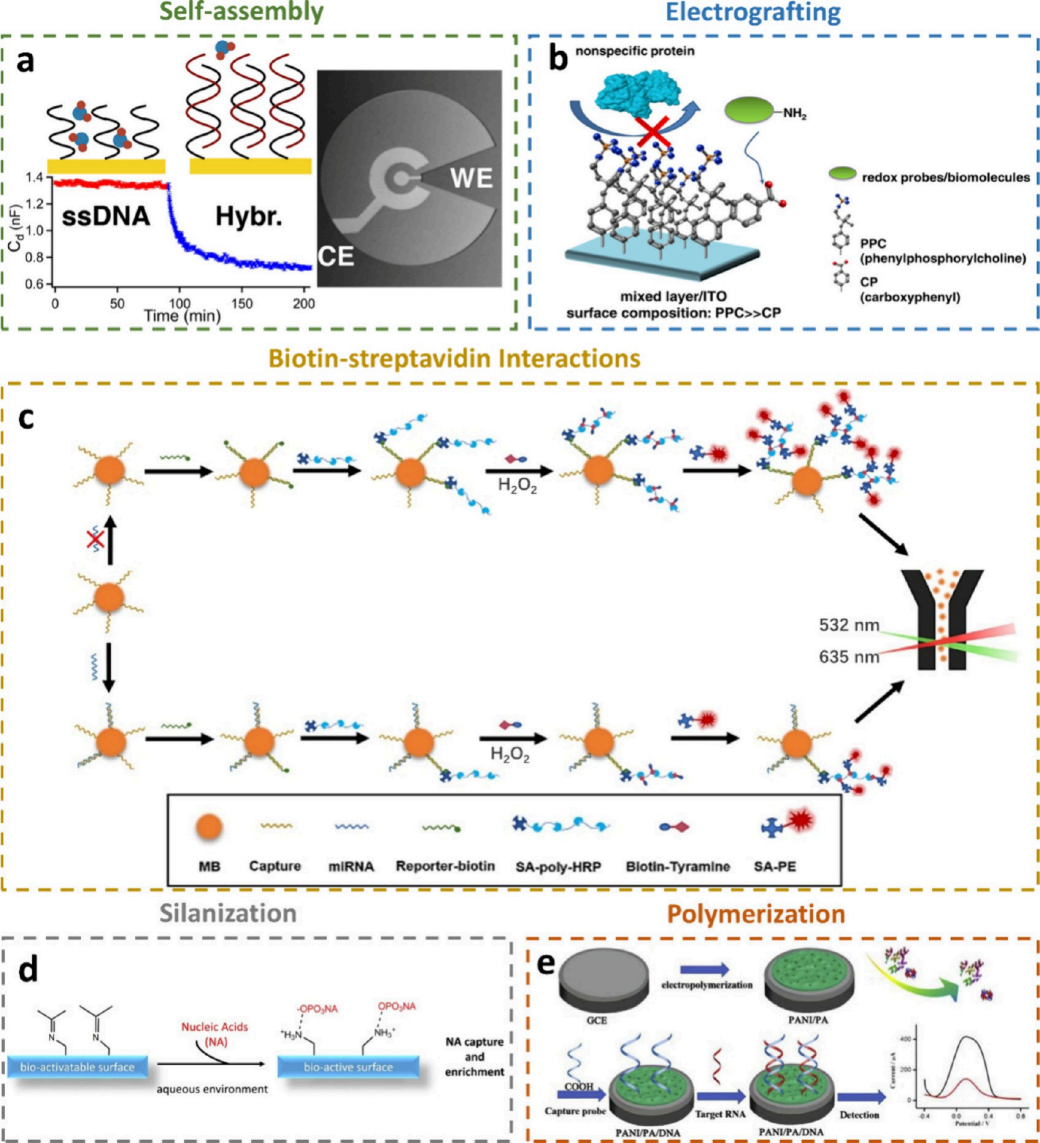
Surface chemistry and bioreceptor immobilization techniques. a) Self-assembly. Reprinted from ref (98). Copyright 2016 American Chemical Society. b) Electrografting. Reprinted from ref (99). Copyright 2016 American Chemical Society. c) Biotin–streptavidin interactions. Reprinted from ref (100). Copyright 2023, with permission from Elsevier. d) Silanization. Reprinted from ref (101). Copyright 2019, with permission from Elsevier. e) Polymerization. Reprinted from ref (102). Copyright 2020, with permission from Elsevier.

The sensitivity and selectivity of electrochemical biosensors largely depend on the efficient immobilization of bioreceptors on the sensor’s surface.^97^ Advances in surface chemistry have led to the development of various methods to immobilize bioreceptors such as antibodies, aptamers, or nucleic acid probes that specifically bind to target miRNAs. These methods include self-assembly,^98^ electrografting,^99^ biotin–streptavidin interactions,^100^ silanization,^101^ and polymerization^102^ (Figure 4). The choice of immobilization strategy affects not only the orientation and density of the recognition molecules but also the stability and repeatability of the sensor.

### Integration of Microfluidics with Electrochemical Biosensors

**Figure 5:**
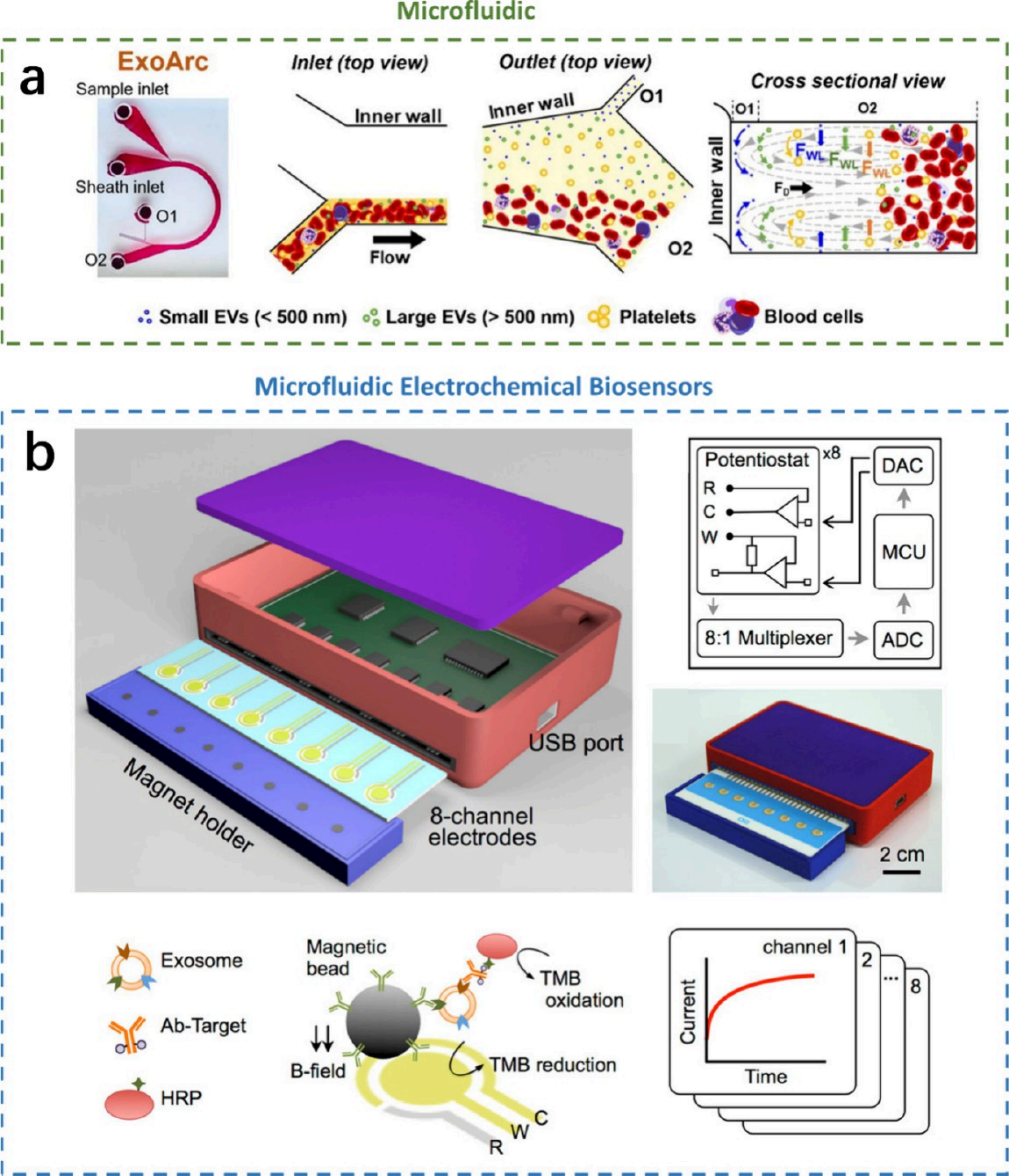
Integration of microfluidics with electrochemical biosensors. a) Microfluidic. Reprinted from ref (103). Copyright 2024 American Chemical Society. b) Microfluidic electrochemical biosensors. Reprinted from ref (104). Copyright 2016 American Chemical Society.

Microfluidics technology involves the control and manipulation of fluids at the microscale, and their integration into electrochemical biosensors has revolutionized their capabilities.^102^ This combination has led to the development of microfluidic electrochemical biosensors, which offer precise fluid handling, reduced sample volumes, and the execution of multiple analytical processes like isolation lysis detection in a single integrated platform.^103^ These sophisticated devices are beneficial for the detection of exosomal miRNAs due to their enhanced sensitivity and specificity, rapid analysis time, and ability to process complex biological samples, such as blood and CSF, with minimal pretreatment. As shown in Figure 5, microfluidics facilitates the specific capture and separation of exosomes from other extracellular vesicles, thereby enhancing the purity and concentration of the exosomal miRNA sample before it reaches the detection chamber.^104^ Furthermore, microfluidic technologies allow for the integration of multiple biosensing elements, which can simultaneously detect a variety of exosomal miRNA biomarkers. This multiplexing capability is crucial for profiling the complex miRNA signatures characteristic of neurodegenerative diseases.^105^ In addition, microfluidics enables on-chip sample preparation, reagent mixing, reaction incubation, and signal detection, all of which contribute to the automation and miniaturization of biosensor devices.

### Signal Processing and Data Analysis Methods

**Figure 6:**
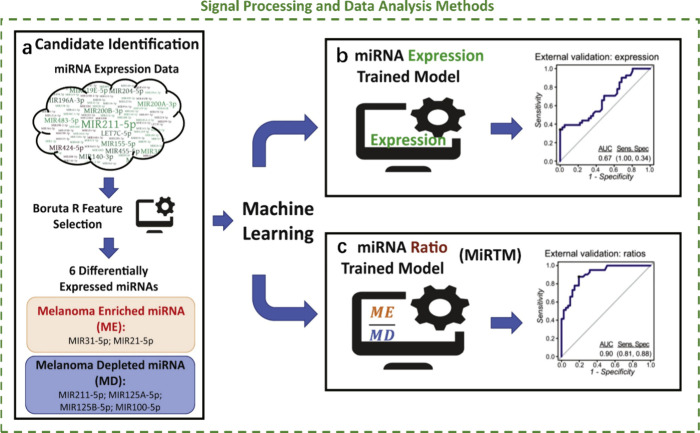
Signal processing and data analysis methods. a) Candidate identification; b) miRNA expression trained model; and c) miRNA ratio trained model (MiRTM). Reprinted from ref (106). Copyright 2020, with permission from Elsevier.

The interpretation of the data collected from electrochemical biosensors is just as critical as the detection technique itself. Advances in signal processing and data analysis methods have greatly contributed to the accuracy and reliability of exosomal miRNA detection. Sophisticated algorithms and computational models^103^ are now used to filter noise, enhance signal quality, and discriminate between the target miRNA and nonspecific interactions. Machine learning techniques^106,107^ have been applied to improve the selectivity and classification of miRNA patterns, allowing for the discernment of disease-specific signatures from complex data sets (Figure 6). Machine learning is applied in two steps: candidate identification (feature selection, Figure 6a) and classification models (Figure 6b and 6c). For the classification models, two different machine learning classifiers were trained, namely, the miRNA expression trained model (Figure 6b) and the miRNA ratio trained model (Figure 6c), and human design of the training strategy can help improve the prediction accuracy of machine learning algorithms. Artificial intelligence algorithms^108^ can analyze vast amounts of biosensor data to identify correlations and trends, which would be impossible to recognize using conventional analysis techniques, matching well with the requirement and scope of the convergence of biotechnology and information technology (BT and IT).

## Applications in Neurodegenerative Diseases

The diagnostic potential of electrochemical biosensors for identifying exosomal microRNA (miRNA) biomarkers has significant relevance in the context of neurodegenerative diseases. This section explores the application of these biosensors in deciphering specific miRNA signatures for Alzheimer’s disease (AD) and Parkinson’s disease (PD), evaluates their sensitivity and specificity, and discusses the challenges ahead for clinical adoption.

### Alzheimer’s Disease

**Figure 7:**
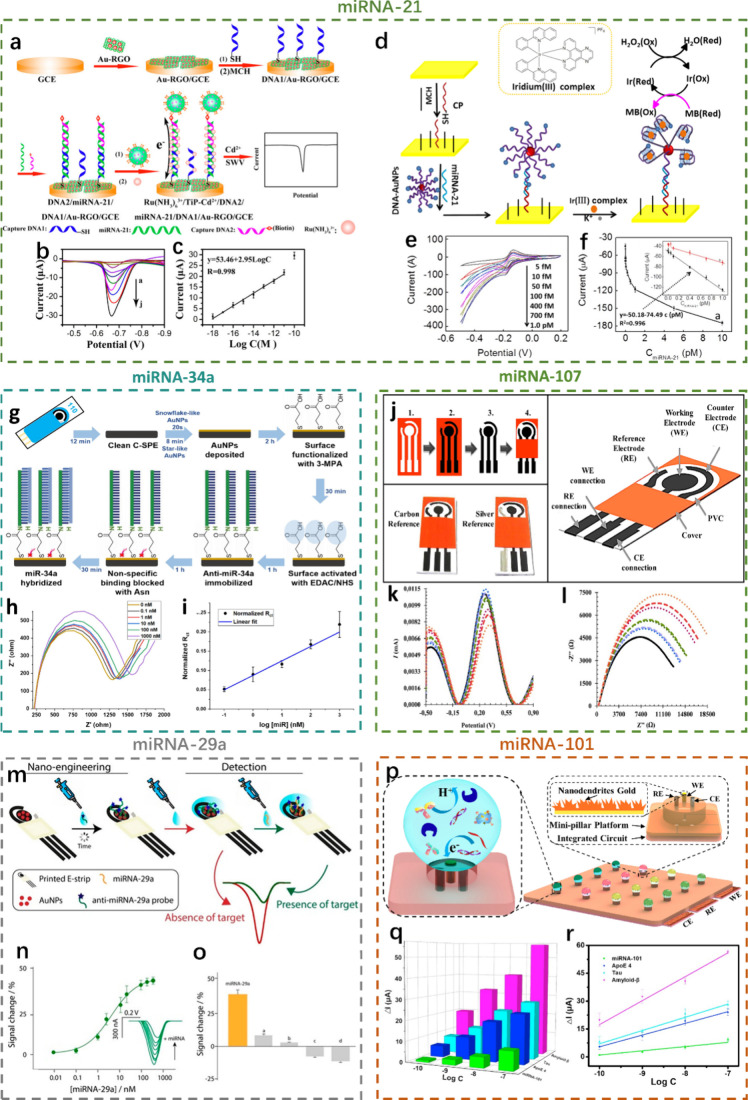
Electrochemical biosensors in Alzheimer’s disease. miRNA-21 as biomarkers: a) DNA-modified AuNPs-rGO/GCE biosensor for miRNA-21 detection; b) square wave voltammetry of the modified AuNPs-RGO/GCE biosensor with increasing miRNA-21 concentration from 0 to 10^–10^ M; and c) linear relationship between the logarithm of the target miRNA-21 and the peak current. Reprinted from ref (112). Copyright 2015 American Chemical Society. d) DNA-modified AuNPs/Au biosensor for miRNA-21 detection; e) cyclic voltammetry of the DNA-modified AuNPs-RGO/GCE biosensor with increasing miRNA-21 concentration from 5 fM to 1 pM; and f) dose–response curve of the target miRNA-21 and the peak current; inset, linear relationship between the target miRNA-21 and the peak current. Reprinted from ref (115). Copyright 2016, with permission from Elsevier. miRNA-34a as biomarkers: g) anti-miRNA-34a-modified AuNPs/SPE biosensor for miRNA-34a detection; h) electrochemical impedance spectroscopy of the anti-miRNA-34a-modified AuNPs/Au biosensor with increasing miRNA-34a concentration from 0 to 1000 nM; and (i) linear relationship between the logarithm of the target miRNA-34a and the charge transfer resistance. Reprinted from ref (116). Copyright 2023, with permission from Elsevier. miRNA-107 as biomarkers: j) SPCE biosensor for miRNA-107 detection; k) square wave voltammetry of the SPCE biosensor with increasing miRNA-107 concentration from 10^–12^ to 10^–6^ M; and l) electrochemical impedance spectroscopy of the SPCE biosensor with increasing miRNA-107 concentration from 10^–12^ to 10^–6^ M. Reprinted from ref (118). Copyright 2018, with permission from Elsevier. miRNA-29a as biomarkers: m) anti-miRNA-29a-modified AuNPs/SPCE biosensor for miRNA-29a detection and n) dose–response curve of the target miRNA-29a and the peak current; inset, square wave voltammetry of the anti-miRNA-29a-modified AuNPs/SPCE biosensor with increasing miRNA-29a concentration from 0.01 to 500 nM. o) Selectivity studies of miRNA-29a. Reprinted from ref (119). Copyright 2022 American Chemical Society. miRNA-101 as biomarkers: p) AuNDs/Au biosensor for miRNA-101 detection; q) electrochemical response and the corresponding calibration curves of miRNA-101, ApoE4, Tau, and Amyloid-β; and r) linear relationship between the peak current and the logarithm of the target biomarkers (miRNA-101, ApoE4, Tau, and Amyloid-β). Reprinted from ref (120). Copyright 2020, with permission from Elsevier.

Alzheimer’s disease is characterized by the presence of amyloid-beta (Aβ) plaques and tau tangles in the brain, which correspond to alterations in certain exosomal miRNAs.^109^ According to proliferation studies, miRNA regulates the phosphorylation of Aβ plaques and tau. Electrochemical biosensors have been employed to detect these miRNA biomarkers associated with pathological pathways in AD.^110^ Case studies highlight the use of electrochemical biosensors to capture miRNA, for instance, which are implicated in tau phosphorylation. The introduction of nanomaterials in screened printed carbon electrodes (SPCE) may provide lower detection limits.^109^ At present, the detection of miRNAs associated with AD is mainly focused on miRNA-21 and miRNA-34a.^111^ Cheng et al. prepared ultrasensitive and highly specific DNA-modified gold nanoparticles, reduced graphene oxide, and a glassy carbon electrode (AuNPs-rGO/GCE) biosensor for miRNA-21 detection, which was developed based on the Cd^2+^-modified titanium phosphate nanoparticle (Cd^2+^-TiPNP) signal unit and the Ru(NH_3_)_6_^3+^ electron transfer mediator to realize amplification and enhanced electron transfer (Figure 7a).^112^ This approach was used between the square wave voltammetry (SWV) peak currents and the logarithm of the target miRNA-21 concentration in a linear range from 1.0 aM to 10.0 pM with an ultralow limit detection of 0.76 aM (Figure 7b and 7c). There is a team that detects miRNA-21 by chronoamperometry (CA). Liu et al. presented a label-free and highly sensitive electrochemical biosensor for miRNA-21 detection by the alkaline phosphatase (ALP) and *p*-aminophenol (p-AP) redox pairs.^113^ Under the optimal experimental conditions, the current increased linearly with the miRNA-21 concentration over a range of 10 fM to 5 pM, and a detection limit of 3 fM was achieved. Unlike the previous method of detecting miRNA-21 by using redox pairs in solution to realize the output of the electrical signal, Shuai et al. realized the output of the electrical signal by deconstructing the hairpin of the hairpin DNA identifying the miRNA-21.^114^ As a result, the electrochemical biosensor can detect target miRNA-21 down to 0.05 fM with a linear range from 0.1 fM to 100 pM. However, Miao et al. simply modified DNA on the gold electrode to quantify miRNA-21 by cyclic voltammetry (Figure 7d) and achieved a detection limit of 1.6 fM for miRNA-21 in the detection range of 5.0 fM to 1.0 pM (Figure 7e and 7f).^115^ Unlike the detection of miRNA-21, the detection of miRNA-34a is mostly achieved by measuring the impedance. As shown in Figure 7g, the quantification of miRNA-34a is achieved by measuring the charge transfer resistance (R_ct_) obtained by data fitting by measuring the impedance (Figure 7h and 7i).^116,117^

In addition to miRNA-21 and miRNA-34a, which are popular for early diagnosis of AD, miRNA-107, miRNA-29a, and miRNA-101 have also been studied by many investigators. Carneiro et al. presented the construction of the SPCE on polyvinyl chloride (PVC) supports and their preliminary testing in the detection of miRNA-107 on a carbon support with a detection limit of 7.08 pM in the detection range of 0.01 to 1000 nM (Figure 7j–7l).^118^ Miglione et al. achieved the detection of miRNA-29a within the detection range of 0.15 to 0.2 nM by modifying anti-miRNA-29a as the identification unit detection miRNA-29a on SPCE with AuNPs (Figure 7m–7o).^119^ It is more interesting that Song et al. demonstrate the mini-pillar-based individual electrochemical array that confines the reagent in open-channel microreactors for simultaneously sensing multiple biomarkers (such as miRNA-101, ApoE4, Tau, and Aβ) (Figure 7p–7r).^120^ The advantage of detecting miRNA-101 over ApoE4, tau, and Aβ is that miRNA-101 exists in exosomes, which can be obtained easily in most body fluids. However, ApoE4, tau, and Aβ are mainly obtained from cerebrospinal fluid. In order to obtain this detection marker, patients need to undergo painful extractions

In conclusion, sensitivity enhancements using nanomaterial-based electrodes provide the low detection limits necessary for these lowly expressed miRNAs, revealing their potential as early AD diagnostic tools.

### Parkinson’s Disease

**Figure 8:**
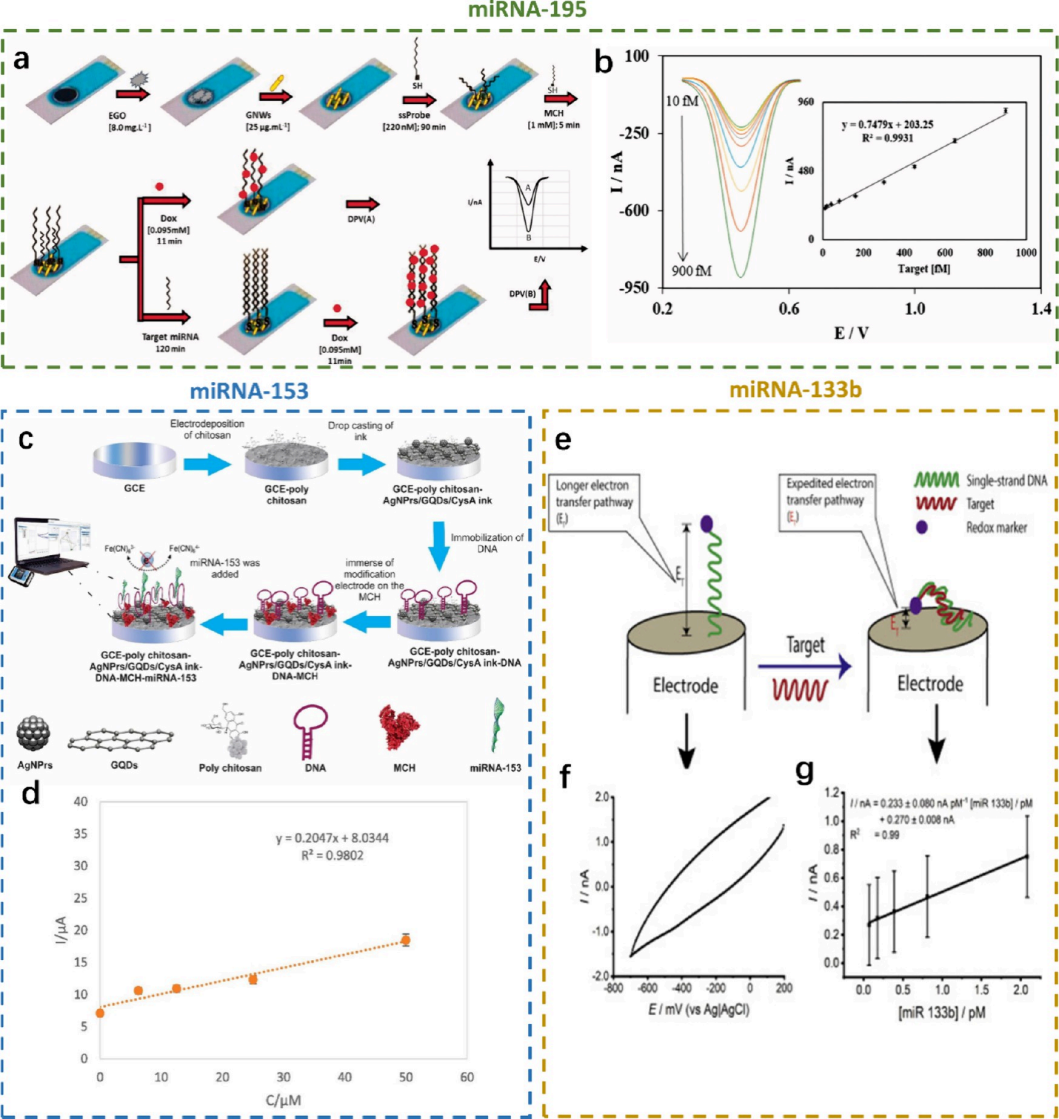
Electrochemical biosensors in Parkinson’s disease. miRNA-195 as biomarkers: a) probe-modified EGO-AuNWs/C biosensor for miRNA-195 detection and b) differential pulse voltammetry of the probe-modified EGO-AuNWs/C biosensor with increasing miRNA-195 concentration from 10 to 900 fM; inset, linear relationship between the peak current and the target miRNA-195. Reprinted from ref (122). Copyright 2018, with permission from Taylor & Francis. miRNA-153 as biomarkers: c) DNA-modified AgNPs-GQDs-CysA/GCE biosensor for miRNA-153 detection and d) linear relationship between the peak current and the target miRNA-153. Reprinted from ref (123). Copyright 2022, with permission from Elsevier. miRNA-133b as biomarkers: e) DNA-modified Au biosensor for miRNA-133b detection; f) cyclic voltammetry of the DNA-modified Au biosensor with miRNA-133b; and g) the linear relationship between the peak current and the target miRNA-133b. Reprinted from ref (124). Copyright 2020, with permission from Elsevier.

For Parkinson’s disease, with its complex pathophysiology involving the loss of dopaminergic neurons, specific miRNAs such as miRNA-195, miRNA-153, and miRNA-133b have been studied (Figure 8).^121^ Electrochemical biosensors support the hypothesis that differentially expressed miRNAs in patient exosomes reflect PD’s neurodegenerative processes. An illustrative case study showed the utilization of carbon-based electrochemical biosensors for detecting miRNA-195 (Figure 8a and 8b).^122^ Aminabad et al. modified the hairpin DNA on silver nanoparticles, graphene quantum dots, and cysteamine A (AgNPs-GQD-CysA) biosensors to detect miRNA-153 within the detection range of 6.25–50 μM (Figure 8c and 8d).^123^ Chandra et al. showed that utilizing graphene-based impedimetric biosensors for detecting miRNA-133b offered a promising approach for early stage PD diagnosis (Figure 8e–8g).^124^ Furthermore, the excellence of the developed biosensing mechanism stems from a simple and cost-effective manufacturing process. As a result, it can be recommended to laboratories and medical experts for use in the early detection of PD, allowing them to accept the disease’s course and potentially treat it when it is appropriate.

### Other Neurodegenerative Diseases

**Figure 9:**
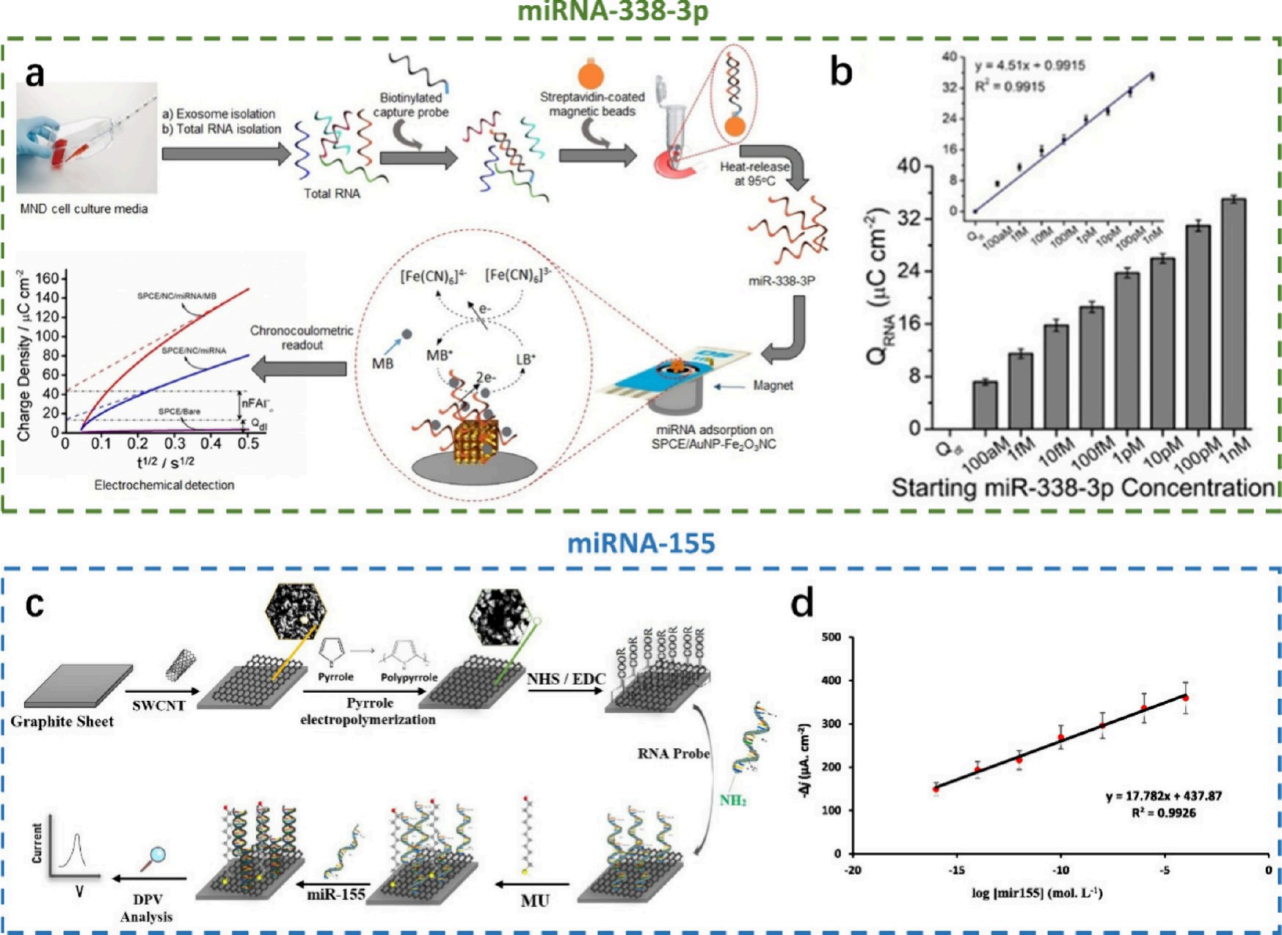
Electrochemical biosensors in motor neuron disease. miRNA-338-3p as biomarkers: a) AuNPs-Fe_2_O_3_NCs/SPCE biosensor for miRNA-338-3p detection and b) dose–response of the target miRNA-338-3p and the charge; inset, linear relationship between the target miRNA-338-3p and the charge. Reprinted from ref (125). Copyright 2020 Wiley. miRNA-155 as biomarkers: c) schematic stages of biosensor fabrication and detection of miRNA-155 and d) calibration curves at different concentrations of miRNA-155. All measurements were performed at 0.01 M PBS (pH 7.4) in the presence of a 5 mM Fe(CN)_6_^–3/–4^ redox probe (*n* = 3). Reprinted from ref (126). Copyright 2022, with permission from Elsevier.

In addition to the two common NDDs, AD and PD, the research on motor neuron disease (MND) and multiple sclerosis (MS) is also exciting.^2^ A group of ailments known as “motor neuron diseases” cause the motor nerves in the spine and brain to gradually become dysfunctional. MND is a very rare severe type of NDD.^2^ Masud et al. reported the electrocatalytic activity of AuNP-Fe_2_O_3_NCs-SPCE toward the redox pairs to achieve the ultrasensitive detection of MND-specific exosomal miRNA-338-3p (Figure 9a).^125^ Under the optimal experimental conditions, the charge increased linearly with the exosomal miRNA-338-3p concentration over the range of 0.1 fM to 1 nM, and a detection limit of 100 aM was achieved (Figure 9b and 9c). However, MS is an autoimmune disease characterized by the breakdown of myelin surrounding the central nervous system (CNS) neurons. It is an autoimmune disease that results in the CNS sclerosis myelin degrading surrounding neurons. Sepideh et al. realized a biosensor comprises a nanocomposite of single-walled carbon nanotubes and polypyrrole on the graphite sheet substrate which was modified with an aptamer as an miRNA-155 capture probe (Figure 9d).^126^ The electrochemical measurements were performed in the presence of Fe(CN)_6_^3–/4–^ as a redox probe, and the biosensor has a dynamic range of 10 aM to 1 μM with a detection limit of 10 aM (Figure 9e). Unlike all of the conventional electrochemical biosensors mentioned above, Macchia et al. achieved extremely low detection limits with organic thin-film transistor (OTFT) biosensors. As a result, the electrochemical biosensor can detect target exosomal miRNA-338-3p down to 10 zM with a linear range from 0.1 to 1000 zM.^127^ Thus, the introduction of transistors may lead to an emerging field of exploration for electrochemical biosensors, providing lower detection limits for biomarkers (i.e., less sample is required for biosensing).

The biological roles of miRNAs, which range from malignant growth and cell division to death, have demonstrated their widespread application in the rapid detection of biosensors. The technique used in electrochemical biosensors, on the other hand, is well suited to the electrophysio-pathological processes of the nervous system and has the potential to significantly alter the early diagnosis and treatment of neurodegenerative illnesses such as AD, PD, MND, and MS, as shown in Table 1.

**Table 1:** Sensitivity, Specificity, and Diagnostic Performance of Current Models^a^

**Disease**	**Target**	**Electrode**	**Nanomaterial**	**Detection method**	**Linear range**	**LOD**	**Ref**
**AD**	miRNA-15a	SPE	MBs	LSV	0.5–3 μg mL^–1^	11.4 nM	(128)
	miRNA-16	SPE	MBs	DPV	5–100 μg mL^–1^	1.4 μM	(129)
	miRNA-21	Au	AuNPs	CV	5–1000 fM	1.6 fM	(115)
	miRNA-21	Au	AuNPs	CA	0.01–5 pM	3 fM	(113)
	miRNA-21	GCE	AuNPs-rGO	SWV	0.001–10000 fM	0.76 aM	(112)
	miRNA-21	GCE	AuNPs-WO_3_-Gr	DPV	0.0001–100 pM	0.05 fM	(114)
	miRNA-29a	SPCE	AuNPs	SWV	0.1–1000 nM	0.15 nM	(119)
	miRNA-34a	PGE	GO	EIS	0.1–10 mg mL^–1^	1.9 mg mL^–1^	(116)
	miRNA-34a	PGE	GO	DPV	5–35 μg mL^–1^	7.5 μg mL^–1^	(130)
	miRNA-34a	SPE	AuNPs	DPV	25–100 μg mL^–1^	10.9 μg mL^–1^	(131)
	miRNA-34a	PGE	GO	EIS	355–2130 nM	261.7 nM	(117)
	miRNA-101	Au	AuNDs	SWV	0.1–100 nM	91.4 pM	(120)
	miRNA-107	SPCE	-	EIS	0.01–1000 nM	7.08 pM	(118)
	miRNA-137	SPCE	erGO-AuNWs	DPV	5–750 fM	1.7 fM	(132)
	miRNA-146a	Au	-	EIS	0.01–1000 nM	10 pM	(133)
	miRNA-1306	Ni	G	EIS	0.1–1000 pM	0.8 fM	(83)
**PD**	miRNA-133b	Au	-	CV	0.01–520 pM	168 aM	(124)
	miRNA-153	GCE	AgNPs-GQDs-CysA	DPV	6.25–50 μM	-	(123)
	miRNA-195	C	eGO-AuNWs	DPV	10–900 fM	2.9 fM	(122)
**Other NDD**	miRNA-182	Au	-	OTFT	0.1–1000 zM	10 zM	(127)
	miRNA-222	SPCE	MBs	DPV	0–1 nM	7 pM	(134)
	miRNA-338-3p	SPCE	AuNPs-Fe_2_O_3_NCs	CC	0.0001–1000 pM	100 aM	(125)

a AD, Alzheimer’s disease; PD, Parkinson disease; NDD, neurodegenerative diseases; SPE, screen-printed electrode; GCE, glassy carbon electrode; SPCE, screen-printed carbon electrode; PGE, pencil graphite electrode; MBs, magnetic beads; NPs, nanoparticles; NDs, nanodendrites; NWs, nanowires; NCs, nanoclusters; G, graphene; GO, graphene oxide; rGO, reduced graphene oxide; erGO, exfoliated reduced graphene oxide; eGO, exfoliated graphene oxide; GQDs, graphene quantum dots; C, carbon; LSV, linear sweep voltammetry; DPV, differential pulse voltammetry; CV, cyclic voltammetry; CA, chronoamperometry; SWV, square wave voltammetry; EIS, electrochemical impedance spectroscopy; OTFT, organic thin-film transistor; CC, chronocoulometric charge.

The performance of electrochemical biosensors is primarily assessed by their sensitivity and specificity. Sensitivity pertains to the ability of the biosensor to detect small quantities of the target miRNA, which is critical given that miRNA levels in exosomes can be extremely low, particularly in the early stages of diseases. Specificity relates to the sensor’s capacity to distinguish the target miRNA from other similar sequences. Current models have achieved sensitivity in the femtomolar range with innovations such as nanocomposite materials and signal amplification strategies, boosting performance. Specificity is often enhanced by incorporating high-affinity capture probes and utilizing stringent washing procedures postbinding to reduce nonspecific adsorption.^135,136^

## Integration with Point-of-Care Diagnostic Platforms

The development and integration of electrochemical biosensors with point-of-care (POC) diagnostic platforms represent a transformative step in the management of neurodegenerative diseases.^7,77,137^ Such integration is paramount for realizing timely and personalized healthcare, and the following subsections outline the interconnected aspects of this integration.

### Growing Trend of Point-of-Care Diagnostics

With the increasing burden of neurodegenerative diseases and the imperative for early detection, there is a growing trend toward point-of-care diagnostics that can be deployed in varied healthcare settings.^138^ These platforms prioritize near-patient testing, facilitating immediate clinical decisions without the need for extensive laboratory infrastructure.^139^ The POC trend has been accelerated by technological advances in biosensor accuracy and mobile health technologies. When it comes to the detection of exosomal microRNA biomarkers, POC diagnostics offer the promise of on-site, real-time, and noninvasive testing, which is indispensable for monitoring and managing progressive neural disorders.^140^

### Design Considerations for Biosensors in Point-of-Care Settings

The design of electrochemical biosensors for POC settings involves multidimensional considerations. A critical aspect is the selection of materials and technologies that allow for the creation of miniature, low-power, and sensitive detection systems. Equally crucial is the biosensor’s selectivity in complex biological matrices typically found in clinical samples. Moreover, POC biosensors must be designed with user friendliness in mind, requiring minimal sample preparation and allowing for easy interpretation of results by nonspecialists. Finally, robustness and reliability are key considerations, ensuring that the biosensors perform consistently under different environmental conditions and in different patient populations.^141^

### Portability, Ease of Use, and Integration with Existing Medical Infrastructure

The ideal POC electrochemical biosensor should be portable, convenient to use, and easily integrated with the existing medical infrastructure. Portability enables in-field diagnostics, which is vital for patients who have difficulty accessing traditional healthcare facilities. Ease of use is achieved through automated cartridge-based systems or disposable test strips that require minimal technical expertise. For successful integration into medical practice, biosensors must interface seamlessly with electronic medical records and laboratory information systems, ensuring that patient data is recorded accurately and is readily accessible to healthcare providers.^142^

### Impact on Patient Outcomes and Healthcare Systems

The implementation of POC electrochemical biosensors is expected to significantly enhance patient outcomes. By enabling early and regular monitoring of exosomal microRNA biomarkers, such sensors can lead to earlier detection of neurodegenerative diseases, prompt treatment initiation, and potentially improved therapeutics.^143^

## Summary and Outlook

As the field of electrochemical biosensing continues to evolve, several trends and innovative strategies are shaping its future, especially in the context of detecting exosomal microRNA for the early diagnosis of neurodegenerative diseases. These developments promise enhancements in detection capabilities, the inception of comprehensive diagnostic modalities, and fruitful collaborations that could bring these technologies from bench to bedside.

Emerging trends in electrochemical biosensor technology focus on the incorporation of cutting-edge materials, novel transduction mechanisms, and advanced fabrication techniques. The integration of nanomaterials such as graphene oxide, carbon nanotubes, and metallic nanoparticles is enhancing the sensitivity and specificity of biosensors. Additionally, advancements in 3D printing and microfabrication are enabling the creation of more sophisticated sensor architectures with enhanced electronic properties and surface areas for increased biomarker interaction.

The field is also witnessing a surge in digital health integration, where biosensors are being designed to seamlessly interface with smartphones and wearable devices. This integration could revolutionize the monitoring of neurodegenerative diseases by enabling the continuous, real-time tracking of exosomal miRNAs in outpatient settings, leading to more personalized medicine approaches.

The detection capabilities of biosensors are being augmented through the development of new recognition elements with higher affinity and selectivity for exosomal miRNAs. Engineered aptamers and synthetic antibodies as well as CRISPR-Cas-based detection systems are at the forefront of this improvement. The expansion of multiplexing abilities, where a single biosensor can detect multiple miRNAs simultaneously by using physical-/chemical-/biological-coding strategies,^105^ is further advancing the diagnostic power of these tools.

Moreover, improvements in signal amplification strategies, including enzymatic amplification and redox cycling, are set to increase the limits of detection, allowing biosensors to identify even the subtlest changes in miRNA profiles indicative of early stage diseases. Faster pathology detection techniques are desperately needed, especially since many diseases are discovered in their later stages. Exosomal miRNAs as biomarkers can meet this need, despite current constraints, making this a promising area of research.

The next generation of electrochemical biosensors is likely to offer multimodal functionalities, combining electrochemical detection with other diagnostic techniques such as optical sensing, mass spectrometry, and magnetic resonance. This convergence could yield a comprehensive diagnostic tool capable of providing a holistic snapshot of disease states by correlating data across different biomarker types including proteins, lipids, and nucleic acids. We believe that as current detection methods advance, miRNA detection with electrochemical biosensors will be routinely used to generate personalized patient profiles, paving the way for early detection and early treatment.
